# Effect of bioceramic-based and resin-based sealers on postoperative discomfort following root canal therapy: a systematic review and meta-analysis

**DOI:** 10.7717/peerj.18198

**Published:** 2024-10-31

**Authors:** Mansi Supare, Ajinkya M. Pawar, Kashmira Sawant, Dian Agustin Wahjuningrum, Suraj Arora, Firas Elmsmari, Mohmed Isaqali Karobari, Bhagyashree Thakur

**Affiliations:** 1 Conservative Dentistry and Endodontics, Nair Hospital Dental College, Mumbai, Maharashtra, India; 2 School of Information, University of Michigan, Ann Arbor, MI, United States of America; 3 Department of Conservative Dentistry, Faculty of Dental Medicine, Universitas Airlangga, Surabaya, Indonesia; 4 Department of Restorative Dental Sciences, College of Dentistry, King Khalid University, Abha, Saudi Arabia; 5 Department of Clinical Sciences, College of Dentistry, Ajman University, Ajman, United Arab Emirates; 6 Center of Medical and Bio-Allied Health Sciences Research, Ajman University, Ajman, United Arab Emirates; 7 Department of Conservative Dentistry and Endodontics, Saveetha Dental College and Hospitals, Saveetha Institute of Medical and Technical Sciences, Saveetha University, Chennai, Tamil Nadu, India; 8 Department of Dentistry, Division of District Early Intervention Centre, Thane Civil Hospital, Thane, Maharashtra, India

**Keywords:** Pain, Discomfort, Root canal therapy, Bioceramic sealers, Resin-based sealers, Systematic review, Meta-analysis

## Abstract

**Background:**

The aim of this systematic review and meta-analysis was to furnish evidence-based recommendations for the utilization of bioceramic-based and resin-based sealers in clinical endodontics, with a focus on reducing postoperative discomfort.

**Methods:**

The investigation’s methodology was registered on the International Prospective Database of Systematic Reviews (PROSPERO: CRD42022355506) and executed using the 2020 PRISMA protocol. Articles were selected utilizing the PICO technique and applying specific inclusion and exclusion criteria. Articles published between January 2000 and August 2022, PubMed, MEDLINE, and DOAJ were utilized as primary data sources. After the identification of studies, two autonomous reviewers evaluated the titles and abstracts, and data from qualifying studies were extracted.

**Results:**

Nine published studies were included in this analysis. The findings indicate that there were no significant differences in the Visual Analog Scale (VAS) scores between resin-based and bioceramic root canal sealers at intervals of 6 hours, 12 hours, 24 hours, and 48 hours after treatment.

**Conclusion:**

The findings of this systematic review and meta-analysis suggest that after the utilization of bioceramic sealers during root canal therapy, the pain and discomfort levels were not significantly different from those experienced pain after the use of resin-based sealers.

## Introduction

Root canal therapy (RCT) is the principal approach to treating root canal infections (Gulabivala & Ng, 2023). The selection of disinfection procedures and obturation materials may play a crucial role in ensuring treatment efficacy and minimizing complications (Silva et al., 2022). Postoperative discomfort is a commonly reported complication among patients after dental procedures, including root canal therapy (Thakur et al., 2023). Physical trauma during treatment, inflammation, and bacterial extrusion are common causes of postoperative discomfort. Iatrogenic causes such as, the selection of instruments and working length or the root canal sealer, may also contribute to postoperative discomfort (Pawar et al., 2022).

The Likert-type scale is a widely recognized and invaluable instrument used to quantify pain and discomfort experienced by patients after endodontic treatment (Santos-Puerta & Peñacoba Puente, 2022). It consists of two different scales, namely the Visual Analog Scale (VAS) and the Four-Point Pain Scale (Delgado et al., 2018). While using the VAS requires patients to mark the intensity of their pain through a line, the Four-Point Pain Scale provides four different rates for the patient to choose their pain level (Atisook et al., 2021). The reliability and efficacy of both scales in evaluating postoperative pain are two vital indicators for healthcare professionals to determine the appropriate course of treatment and manage patient expectations (Mahama & Ninnoni, 2019). Consequently, the use of Likert-type scales has been an increasingly accepted practice in endodontic therapy and other medical fields. The choice of obturation technique can significantly influence postoperative pain and discomfort, with the cold lateral compaction technique being the most effective in minimizing these issues (Wong et al., 2017).

Several studies have investigated the relationship between root canal sealers and postoperative pain with various results (Ferreira de et al., 2020a; Shim, Jang & Kim, 2021; Monteiro et al., 2023; Coşar, Kandemir Demirci & Çalışkan, 2023). Evidence suggests that specific types of sealers, like resin-based sealers, might be linked to higher levels of postoperative discomfort compared to other sealers, such as zinc oxide eugenol-based sealers (Özdemir & Kopac, 2022). The composition of the root canal sealers may play a key role in managing postoperative pain and discomfort. Bioceramic root canal sealers (BCS) have garnered considerable attention in recent years due to their remarkable sealing ability and biocompatibility. BCS synergistically create a highly bioactive and biocompatible substance by combining calcium silicates, monobasic calcium phosphate, zirconium oxide, filler particles, and a hydrophilic polymer. These components actively support tissue regeneration and effectively inhibit the growth of bacteria, fostering a conducive environment for optimal healing. Numerous studies have demonstrated that BCS can significantly reduce postoperative pain in root canal therapy by inhibiting bacterial growth and promoting tissue healing (Mekhdieva et al., 2021). Compared to patients treated with traditional zinc oxide eugenol-based or resin-based root canal sealers, patients treated with BCS experienced much less postoperative pain. Resin-based sealers have been shown to produce residual monomers and cause cytotoxicity, which can be uncomfortable even when they show good biocompatibility (Javidi et al., 2020). These sealers consist of a combination of resin monomers, fillers such as quartz, silica, glass particles, and zirconium oxide, with additional additives to impart the necessary chemical and physical characteristics (Cho et al., 2022). In some circumstances, resin-based sealers might be suitable, especially if a patient is allergic to any of the chemicals in other kinds of sealers (Syed, Chopra & Sachdev, 2015). Resin sealers can be used to avoid triggering allergic reactions in such cases. Additionally, resin-based sealers are more effective in sealing off microcracks and fissures in the root canal system.

From the root canal issue highlighted, this systematic review and meta-analysis of relevant clinical studies aim to assess and compare the effects of bioceramic-based and resin-based root canal sealers in alleviating/preventing postoperative pain. The secondary aim was to evaluate the consumption of analgesics required to treat postoperative pain associated with the use of either bioceramic or resin-based sealers.

## Survey Methodology

### Registration and protocol

A systematic review of the literature and meta-analysis was performed. This study followed the Preferred Reporting Items for Systematic Review (PRISMA 2020), the Cochrane Handbook for Systematic Reviews of Interventions, version 5.1.0, and the 4th Edition of the JBI Reviewer’s Manual. It was registered to PROSPERO under registration code CRD42022355506.

### Research question

Using the “PICO” (PRISMA 2020) technique, this study sets a research question focusing on the effectiveness of bioceramic-based and conventional resin-based sealers in managing postoperative pain after root canal therapy based on relevant clinical trials.

• **Population:** Individuals receiving non-surgical root canal therapy for their permanent teeth
• **Intervention:** Root canal treatment using bioceramic sealers
• **Comparison:** Root canal treatment using resin-based sealers
• **Outcomes:** Post-operative pain scores and post-operative analgesic use

### Data sources

The PICOS criteria were applied to screen potential research articles. Titles and abstracts were assessed independently by two reviewers; any discrepancies were discussed with a third reviewer. The Directory of Open Access Journals (DOAJ) and PubMed MEDLINE were among the electronic resources evaluated. Using precise keywords and MeSH phrases combined with Boolean operators, a thorough search was conducted that included articles published between January 2000 and August 2022, without language constraints (Table 1).

**Table 1:** Medical Subject Headings (MeSH) keywords used in literature search.

**Databases**	**Search strategy**	**Results**
**PubMed**	((bioceramic [All Fields] AND sealers [All Fields]) AND (conventional [All Fields] AND sealers [All Fields])) AND (”pain, postoperative”[MeSH Terms] OR (”pain” [All Fields] AND ”postoperative” [All Fields]) OR ”postoperative pain” [All Fields] OR (”post” [All Fields] AND ”operative” [All Fields] AND ”pain” [All Fields]) OR ”post-operative pain” [All Fields])	2,279
**Google Search**	Bioceramic sealer, Resin based sealers Post-operative pain	1,673
**Embase**	(“bioceramics” AND “sealers” OR “conventional sealers” AND “postoperative pain” OR “operative pain” OR “post-operative pain”)	1,286
**Scopus**	(“Bioceramics” OR “bioceramics” OR “biosealers”) AND (“sealers” OR “conventional sealers”) AND (“post-operative pain” OR “postoperative pain”)	1,191
**Hand Search**	Australian Endodontic Journal, Iranian Endodontic Journal, International Endodontic Journal, and Journal of Endodontics	32
**Total**		6461

### Eligibility criteria

Research published between January 1, 2000, and August 30, 2022, that involved subjects receiving non-surgical root canal therapy on permanent teeth utilizing bioceramic and resin-based sealers were considered. A third reviewer arbitrated disagreements between the two reviewers after they had used the PICOS technique to assess entire texts and create inclusion and exclusion criteria (Table 2).

**Table 2:** Inclusion and exclusion criteria.

**Inclusion criteria**	• Studies focused on participants with non-surgical root canal treatment done on permanent teeth in a single visit • Studies involving root canal treatment done using bioceramic sealers or resin-based sealers; studies giving information about mean post-operative pain scores; studies giving information about the quality of obturation including underfill, optimal fill, or overfill; studies published in English only • Studies published between January 2000 and August 2022 • Studies using randomized controlled trials, prospective clinical trials, and quasi-experimental study designs • Full-text articles • Studies comparing results to a control group or to a valid reference standard
**Exclusion criteria**	• Studies involving patients who do not provide informed consent • Studies involving other sealer systems • Studies using any comparison group other than resin-based sealers • Review reports, case series, in-vitro, and animal studies • Studies having only abstracts and not full texts

### Selection of studies

Two independent reviewers (M.S. and A.M.P.) conducted a critical assessment of the title and abstract of each study. The selection process involved:

(i) Removing duplicate entries
(ii) Assessing titles and abstracts to exclude irrelevant articles
(iii) Retrieving full texts of potentially relevant articles
(iv) Ensuring a comprehensive collection of relevant information
(v) Full-text examination for eligibility criteria compliance
(vi) Consulting researchers for eligibility clarifications if needed
(vii) Determining inclusion criteria and data collection

### Data extraction

Two reviewers (M.S. and A.M.P.) selected studies and extracted relevant data using a comprehensive checklist, including details like authors, year, study design, sample size, age group, gender, randomization, blinding, outcome assessment, results, and other pertinent data. Discrepancies were resolved through discussion or by consulting a third reviewer (D.A.W.) for final judgment.

Data on publication and study, participants, interventions, comparators, outcome measures, research design, statistical analysis, and findings, as well as any other pertinent data (*e.g.*, funding and conflicts of interest) were methodically collected from all selected studies. Two reviewers performed data extraction, and all primary outcomes were meticulously recorded in separate Excel sheets.

Any differences of opinion between the two independent reviewers (M.S. and A.M.P.) during the selection of articles for the systematic review and meta-analysis were settled by discussion and agreement. When disagreements developed amongst the primary reviewers while selecting articles for inclusion in the systematic review and meta-analysis (SRMA), discussions were held to reach a solution. If consensus could not be reached, a third independent reviewer was entrusted to arbitrate and make a final judgment. The third reviewer (D.A.W.) was chosen for her subject knowledge and functioned as an unbiased adjudicator to settle concerns and ensure the selection process was rigorous and objective.

### Quality assessment

Evaluation of the research quality was performed using the Cochrane Bias Risk-2 (ROB-2), clinical and randomized controlled trials tool, which includes areas such as random sequence generation, allocation concealment, participant blinding, inadequate outcome data, selective reporting, and other biases. Quality assessment was done using Review Manager version 5.4.

### Meta-analysis

Meta-analysis was performed on trials with comparable results and periods between follow-ups. Standard deviations and mean instrumentation times were used to evaluate continuous data. While quantitative synthesis computed a combined estimate of the intervention impact, taking heterogeneity (I2) into account to apply a suitable effect model (fixed or random), descriptive synthesis offered an overview of the main research aspects. Review Manager 5.4 was utilized for conducting quantitative synthesis.

## Results

**Figure 1:**
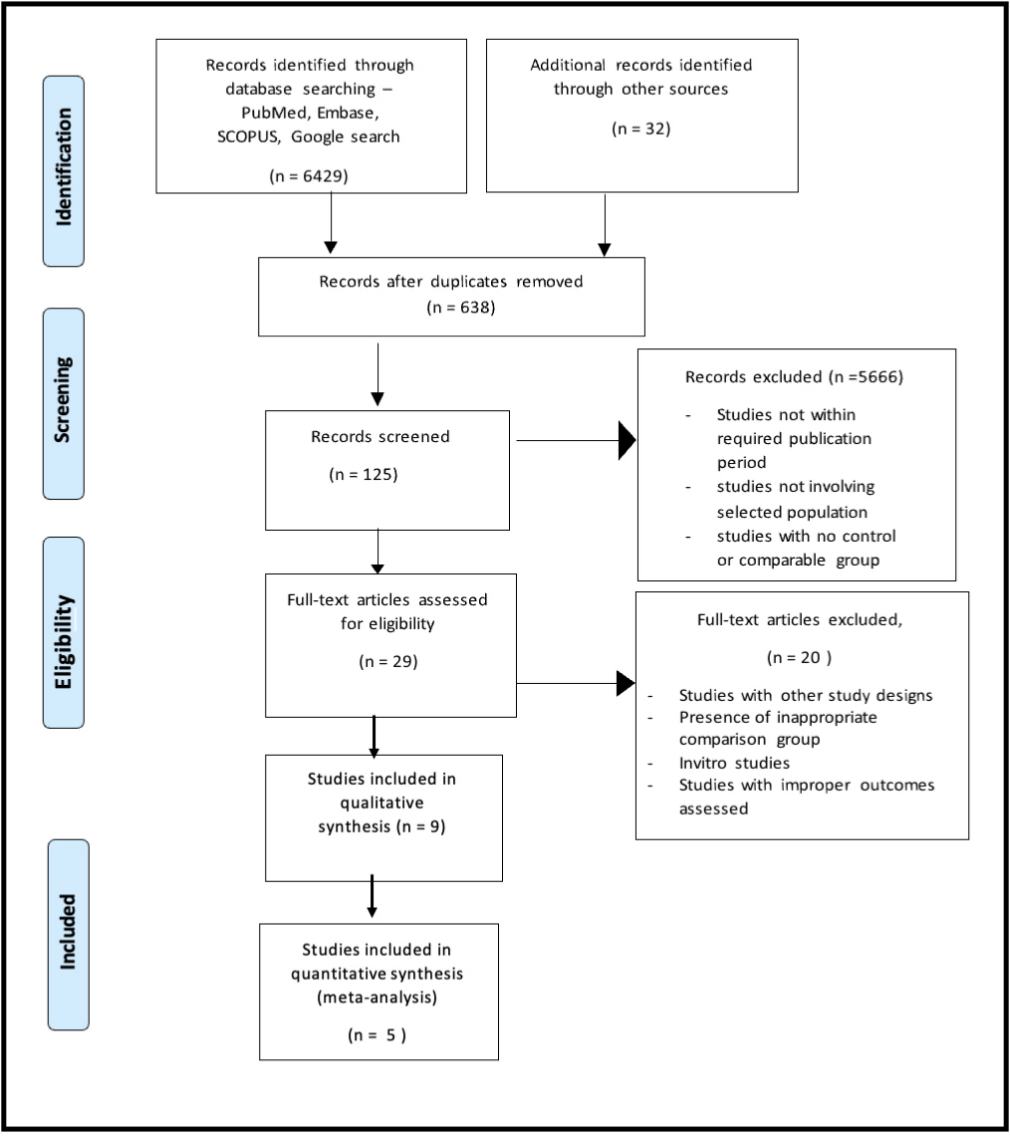
PRISMA flow diagram.

The initial electronic database search yielded 6,461 titles from PubMed/MEDLINE, the Cochrane Library, DOAJ, Embase, SCOPUS and Google search. There were 638 duplicate articles. From the screening of the abstracts, two independent reviewers selected 254 relevant titles, and 5,666 were removed due to having an unrelated topic and publication year. Based on the reviewers’ decision, 19 articles were chosen for full-text evaluation. The manual search of the references from the selected studies yielded no matched articles. Following pre-screening, the articles were selected using inclusion and exclusion criteria. Then, based on the PICO questions, nine studies of the total search were included in the qualitative synthesis or data extraction. Meta-analysis of five selected studies was then conducted (Fig. 1).

### Research characteristics

Nine studies were selected for qualitative synthesis. Their general characteristics are presented in Table 3. All the studies that fulfilled the inclusion criteria of this systematic review were solely randomized controlled clinical trials. In total, 678 participants were involved in the analysis of the selected studies, generating a comprehensive examination of the subject matter. These studies were conducted in all parts of the world. All the studies reached a consistent conclusion that there was no notable distinction in the levels of postoperative pain between the two types of sealers.

**Table 3:** Characteristics of included studies.

**Author**	**Country**	**Study** **design**	**Sample** **size**	**Age** **(years)**	**Intervention** **group**	**Control** **group**	**Follow** **up**	**Outcome** **assessed**	**Conclusion**
Atav Ates et al. (2019)	Turkey	RCT	160	18-65 years	BC sealer	AH plus vital	6, 12, 24, 72 h	Pain, obturation quality	Despite the absence of a significant effect on postoperative pain levels after root canal obturation, the iRoot SP sealer was linked to lower analgesic intake more than AH plus sealer.
Graunaite et al. (2018)	Lithuania	RCT, Split mouth	61	49.5 ± 12.82	Total fill sealer (bioceramic based)	AH plus (resin based)	12, 24, 72 h, 7 days	Post-operative pain	When treatment-related irritants were minimized, the occurrence and intensity of postoperative pain were found to be similar for both sealers.
Fonseca et al. (2019)	Brazil	RCT	64	>18 years	Sealer plus BC	AH plus (resin based)	24, 48 h	Post-operative pain	The RG sealer exhibited significantly less extrusion compared to the BG sealer. However, no significant association was found between sealer extrusion and pain. The average pain intensity and the mean frequency of analgesic use were comparable in both groups.
Sharma et al. (2020)	India	RCT	40	not mentioned	MTA plus	AH plus (resin based)	24, 48 h	Post-operative pain	No significant difference was observed in post-endodontic pain scores between the groups of sealers, indicating that either group of sealers can be used for single-visit endodontics without concerns about postoperative pain.
Ferreira de et al. (2020a); Ferreira de et al. (2020b)	Brazil	RCT	60	<18 years	AH plus (resin based)	Endofill	24, 48 h	Post-operative pain	The utilization of AH Plus, MTA-Fillapex, and Endofill for root canal filling did not lead to any significant difference in terms of postoperative pain occurrence and intensity or the requirement for analgesic intake.
Tan et al. (2021)	Singapore	RCT	163	>21 years	Total fill sealer (bioceramic based)	AH plus (resin-based)	1, 3,7 days	Post-operative pain	After obturation, there was no notable distinction in pain occurrence among teeth filled with AH Plus^®^ or TotalFill^®^ BC sealer after 1, 3, and 7 days. However, various factors linked to the patient and the treatment could have an impact on post-obturation pain.
Shim, Jang & Kim (2021)	China	RCT	67	49.04 ± 16.62	MTA seal	AH plus devital	–	Post-operative pain, obturation time	In this study, it was found that there was no significant difference in postoperative pain incidence and intensity between Endoseal MTA and AH plus sealers. Moreover, obturation time was less in the Endoseal MTA group as compared to the AH plus group.
Khandelwal et al. (2022)	India	RCT	63	–	MTA seal	AH plus devital	24, 48, 72 h, 7 days	Pain, periapical healing	After root canal treatment, the use of BioRoot RCS as the endodontic sealer resulted in less postoperative pain compared to the use of AH plus and Tubli-Seal.
Khatri et al. (2022)	India	Clinical Trial	60	–	MTA-fillapex	AH plus (RB)	7, 24, 48, 72 hours	Post-operative pain	After a single visit to root canal treatment, there was no significant difference found in postoperative pain levels between the use of AH plus, MTA Fillapex, and Sealapex sealers.

The qualitative summary included the research conducted by Atav Ates et al. (2019). In this investigation, 60 patients were randomized to have root canal therapy using either AH Plus, a resin-based sealer, or EnddoSequence BC Sealer, a bioceramic-based sealer. A visual analog scale (VAS) was used to measure postoperative pain at 6-, 12-, 24-, and 48-hours following treatment. The pain levels of the two sealer groups did not differ significantly at any stage during the study, according to the findings.

### Risk of bias applicability

**Figure 2:**
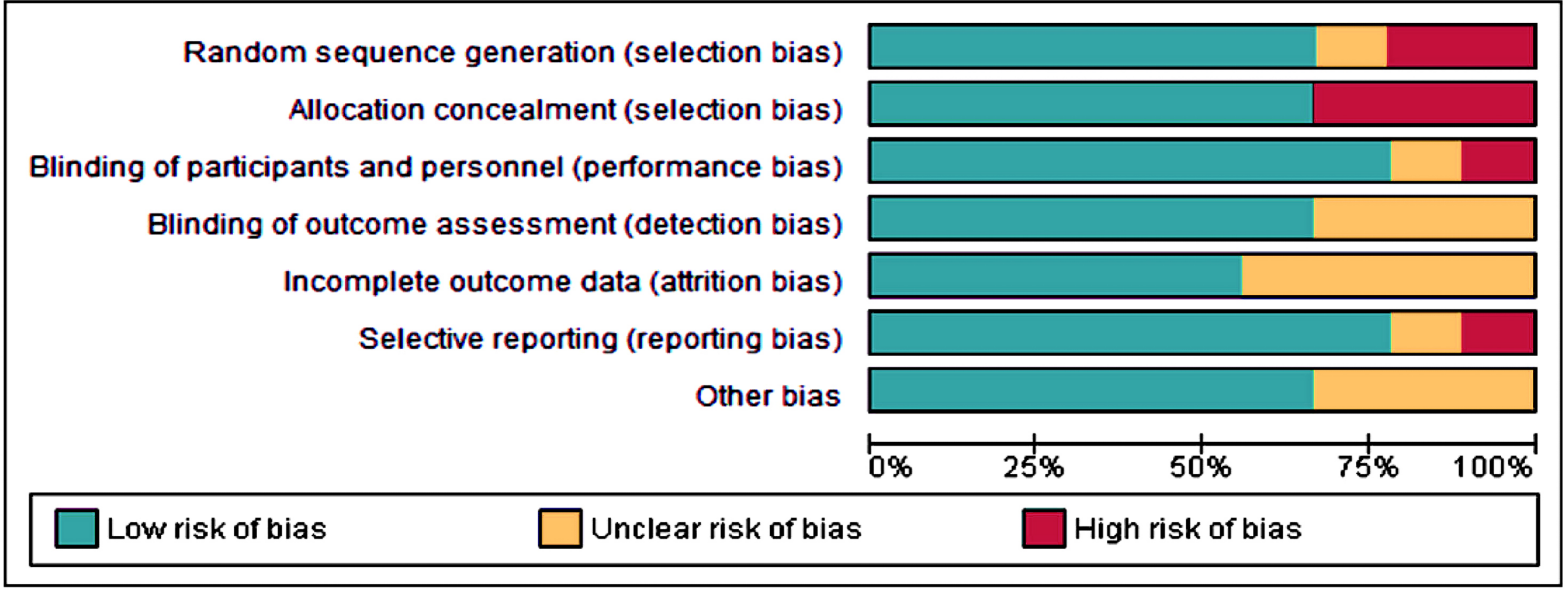
Risk of bias graph of the included studies.

**Figure 3:**
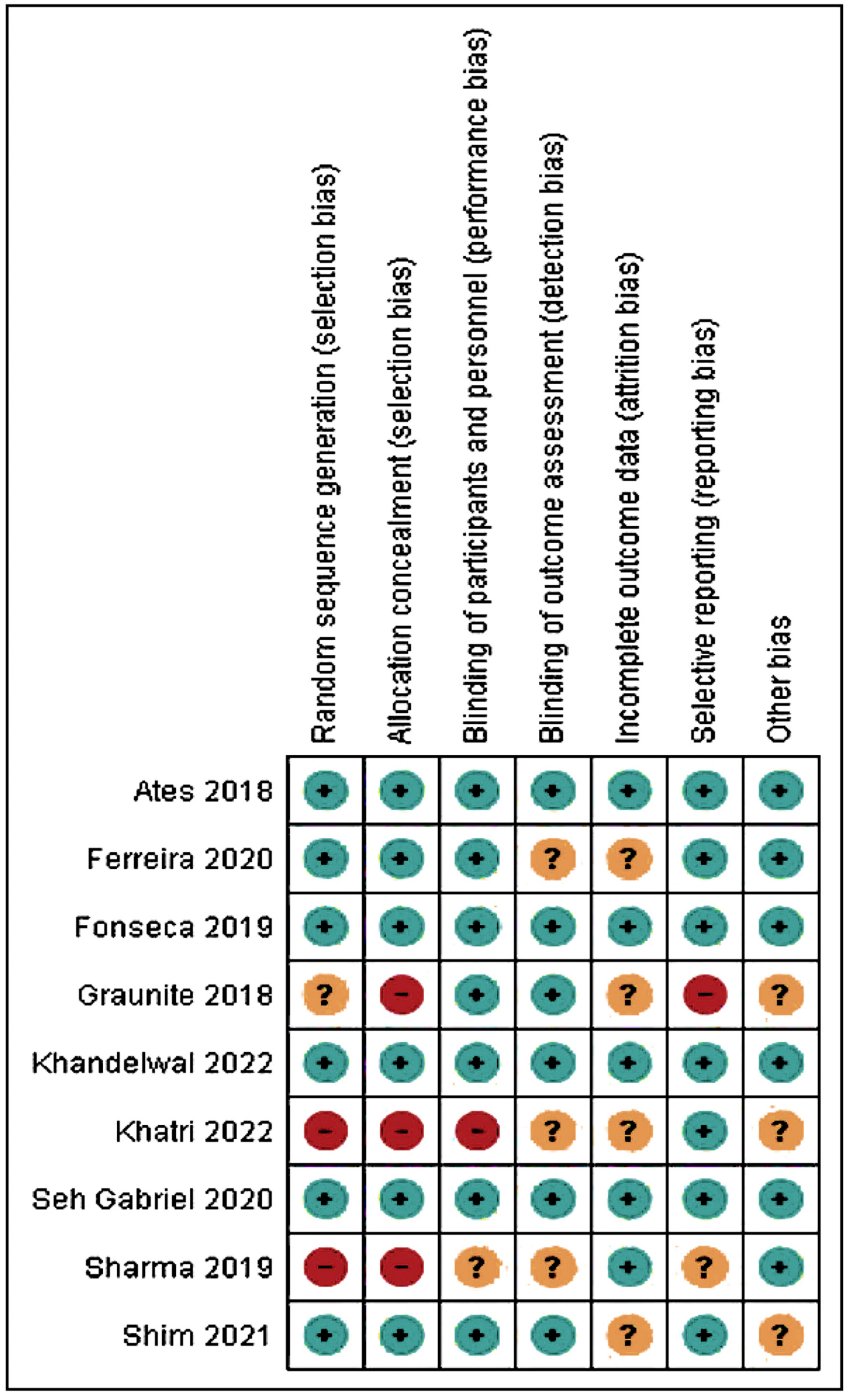
Risk of bias assessment of the included studies.

The Cochrane Risk of Bias Tool (ROB-2) was used for evaluating the quality of randomized controlled trials (Table 4; Figs. 2 and 3). Four studies (Fonseca et al., 2019; Atav Ates et al., 2019; Ferreira de et al., 2020b; Khandelwal et al., 2022) exhibited a low risk of bias; two studies (Ferreira de et al., 2020c; Shim, Jang & Kim, 2021) showed a moderate risk; and three studies (Graunaite et al., 2018; Sharma et al., 2020; Khatri et al., 2022) displayed a high risk of bias. The absence of random sequence generation was not reported in three studies (Graunaite et al., 2018; Sharma et al., 2020; Khatri et al., 2022), contributing to the high risk of bias in these studies. All studies, except for Sharma et al.’s (2020) research, employed blinding techniques.

**Table 4:** Risk of bias assessment.

**Sr.** **No.**	**Author**	**Random** **sequence generation**	**Allocation concealment**	**Blinding of** **participants and personnel**	**Blinding of** **outcome assessment**	**Incomplete outcome data**	**Selective reporting**	**Other bias**	**Risk of bias**
1	Atav Ates et al. (2019)	Yes	Yes	Yes	Yes	Yes	Yes	Yes	Low risk
2	Graunaite et al. (2018)	Unclear	No	Yes	Yes	Unclear	No	Unclear	High risk
3	Fonseca et al. (2019)	Yes	Yes	Yes	Yes	Yes	Yes	Yes	Low risk
4	Sharma et al. (2020)	No	No	Unclear	Unclear	Yes	Unclear	Yes	High risk
5	Ferreira de et al. (2020a); Ferreira de et al. (2020b)	Yes	Yes	Yes	Unclear	Unclear	Yes	Yes	Moderate risk
6	Tan et al. (2021)	Yes	Yes	Yes	Yes	Yes	Yes	Yes	Low risk
7	Shim, Jang & Kim (2021)	Yes	Yes	Yes	Yes	Unclear	Yes	Unclear	Moderate risk
8	Khandelwal et al. (2022)	Yes	Yes	Yes	Yes	Yes	Yes	Yes	Low risk
9	Khatri et al. (2022)	No	No	No	Unclear	Unclear	Yes	Unclear	High risk

### Meta-analysis

Meta-analysis was conducted on the following parameters:

1. Post-operative pain at 6 h, 12 h, 24 h and 48 h
2. Post-operative analgesic use after 24 h.

Six studies (Graunaite et al., 2018; Fonseca et al., 2019; Atav Ates et al., 2019; Tan et al., 2021; Khandelwal et al., 2022; Khatri et al., 2022) were included in the meta-analysis. The statistic test used to quantify the inconsistency (heterogeneity) between studies was the I^2^. The results of the meta-analysis were then interpreted by the Cochrane Handbook for Systematic Reviews of Interventions.

### Effect sizes

Effect sizes serve as quantitative measures that indicate the magnitude and direction of the impact interventions on outcomes. To find differences in continuous data (specifically mean values), effect sizes were calculated using information on the mean response, standard deviation, and number of participants within each group.

### Six-hour post-operative pain level

**Figure 4:**
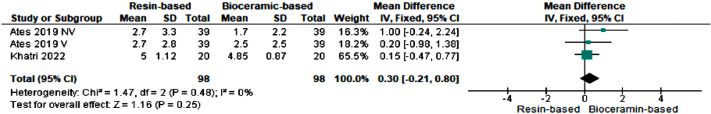
Forest plot for post-operative pain score between the groups after six hours of treatment. Note: See Atav Ates et al. (2019) and Khatri et al. (2022).

Two studies (Atav Ates et al., 2019; Khatri et al., 2022) evaluated post-operative pain after six hours of treatment. The pooled mean difference was 0.30 [−0.21, 0.80] indicating that the mean pain scores were greater with resin-based sealers than with bioceramic sealers at six hours. Heterogeneity (I2) was 0%, and a fixed effect model was used. The results were not statistically significant (*p* > 0.05) (Fig. 4).

### Twelve-hour post-operative pain level

**Figure 5:**
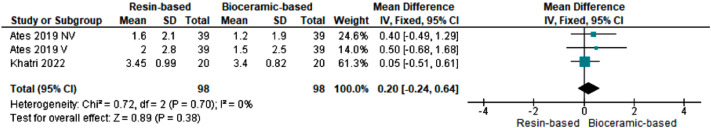
Forest plot for Post-operative pain score between the groups after 12 h of treatment. Note: See Atav Ates et al. (2019) and Khatri et al. (2022).

Two studies (Atav Ates et al., 2019; Khatri et al., 2022) evaluated post-operative pain after 12 h of treatment. The pooled mean difference was 0.20 [−0.24, 0.64] indicating that mean pain scores were greater with resin-based sealers than with bioceramics sealers at 12 h. Heterogeneity (I2) was 0%, and a fixed effect model was used. The results were not statistically significant (*p* > 0.05) (Fig. 5).

### Twenty-four-hour post-operative pain level

**Figure 6:**
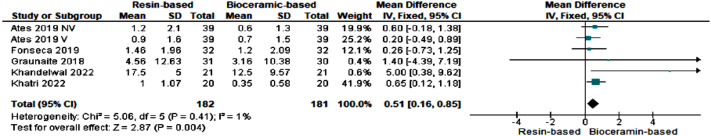
Forest plot for post-operative pain score between the groups after 24 h of treatment. Note: See Atav Ates et al. (2019), Fonseca et al. (2019), Graunaite et al. (2018), Khandelwal et al. (2022) and Khatri et al. (2022).

Five studies (Graunaite et al., 2018; Fonseca et al., 2019; Atav Ates et al., 2019; Khandelwal et al., 2022; Khatri et al., 2022) evaluated post-operative pain after 24 h of treatment. The pooled mean difference was 0.51 [0.16, 0.85] indicating that mean pain scores were greater with resin-based sealers than with bioceramics sealers at 24 h. Heterogeneity (I2) was 1%, and thus a fixed effect model was used. The results were not statistically significant (*p* > 0.05) (Fig. 6).

### Forty-eight-hour post-operative pain level

**Figure 7:**
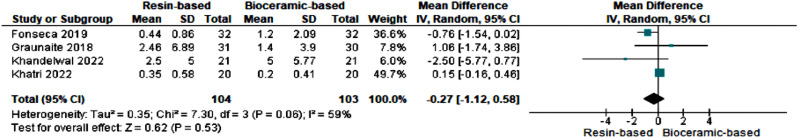
Forest plot for post-operative pain score between the groups after 48 h of treatment. Note: See Fonseca et al. (2019), Graunaite et al. (2018), Khandelwal et al. (2022) and Khatri et al. (2022).

Four studies (Graunaite et al., 2018; Fonseca et al., 2019; Khandelwal et al., 2022; Khatri et al., 2022) evaluated postoperative pain after 48 h of treatment. The pooled mean difference was −0.27 [−1.12, 0.58] indicating that mean pain scores were greater with bioceramics sealers than with resin-based sealers at 48 h. Heterogeneity (I2) was 59%, thereby using a random effect model. The results were not statistically significant (*p* > 0.05) (Fig. 7).

### Analgesic use

**Figure 8:**
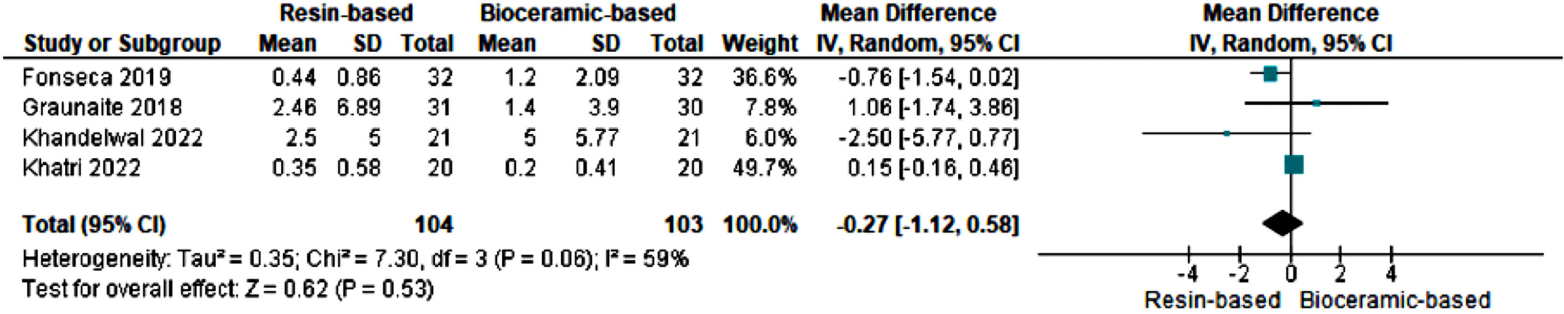
Forest plot comparing the intake of analgesics after 24 h of treatment. Note: See Fonseca et al. (2019), Graunaite et al. (2018), Khandelwal et al. (2022) and Khatri et al. (2022).

Four studies (Graunaite et al., 2018; Fonseca et al., 2019; Atav Ates et al., 2019; Tan et al., 2021) evaluated analgesic use after 24 h of treatment. The pooled risk ratio indicated that the risk of analgesic use with resin-based sealer was 1.89-fold (RR = 1.89 [0.61, 5.81]) more than with bioceramics-based sealer at 24 h of treatment. Heterogeneity (I2) was 59%, thereby utilizing a random effect model. The results were not statistically significant (*p* > 0.05) (Fig. 8).

## Discussion

The present investigation evaluated the effects of two root canal sealers on postoperative pain in single-visit endodontic treatment: resin-based sealers (RBS) and bioceramic-based sealers (BCS). Every study that was included completed the entire root canal procedure—including root canal preparation and obturation in a single visit. Endodontic procedures can induce patient anxiety, especially when complications and pain arise and thus exacerbate this anxiety (Peng et al., 2007; Alroomy et al., 2020). Severe pain after root canal treatment reflects intricate cellular-level physiological changes (Gotler, Bar-Gil & Ashkenazi, 2012). At the start of endodontic therapy, three potential outcomes that may occur include no symptoms, manageable pain/pressure, or intense pain/swelling which needs an unscheduled clinical visit (Bassam et al., 2021).

Post-operative endodontic pain often stems from local inflammatory responses in periapical tissue, with biochemical mediators such as reactive oxygen species (ROS) contributed to the discomfort (Georgiou et al., 2021). Root canal sealers, including bioceramic-based sealers (BCS) and resin-based sealers (RBS), can induce post-operative discomfort by releasing ROS and activating trigeminal nociceptors and pain-sensitive sensory receptors. This activation may lead to increased irritation and discomfort, possibly exacerbated by the release of calcitonin gene-related peptides (CGRP) *via* nociceptor stimulation (Graunaite et al., 2018; Jamali et al., 2021). Therefore, selecting the suitable sealer and applying it correctly is essential to minimize post-operative pain and discomfort.

This study focused on comparing the frequency of postoperative pain associated with two distinct types of root canal sealers: BCS and RBS. BCS, composed of biocompatible and bioactive inorganic materials, offers high sealing qualities, biocompatibility, and antibacterial activity. In contrast, RBS, made of organic components, may exhibit lower biocompatibility and antibacterial activity but its high pH that can neutralize the root canal’s acidic environment and benefit periapical tissues (Wang, 2015; Wang, Shen & Haapasalo, 2021). Notably, resin-based sealers like AH plus may release residual monomers, potentially triggering inflammation and discomfort, while BCS, with its biocompatible and bioactive inorganic materials, ensures a tight seal within the root canal, preventing bacterial entry and infection (Sanz et al., 2022; Torbati et al., 2023). Choosing a certain type of sealer may have a significant impact on postoperative patient comfort after endodontic procedures.

A meta-analysis was conducted to assess postoperative pain levels at specific intervals of 6, 12, 24, and 48 h following dental procedures. The importance of evaluating pain at these particular time points is that it can significantly impact a patient’s comfort, function, and overall quality of life. An accurate understanding of pain duration, intensity and these critical intervals can help clinicians develop effective pain management strategies, minimize patient discomfort and promote optimal healing (Choi et al., 2021).

Post-operative pain at six hours is a key indicator of immediate post-operative discomfort, while pain levels at 12 h signify the patient’s recovery (Garimella & Cellini, 2013). Two studies (Atav Ates et al., 2019; Khatri et al., 2022) compared post-operative pain in patients using resin-based and bioceramic-based sealers at six and 12 h of interval. The pooled mean difference was 0.30, suggesting slightly higher pain scores in resin-based sealers. However, with low heterogeneity (I2 = 0%) and a significance level (*p* = 0.25) greater than 0.05, the difference in pain scores between the two groups may not be substantial enough to cause different levels of pain.

Post-operative pain at 24 h is a crucial marker in a patient’s recovery. Five studies (Graunaite et al., 2018; Fonseca et al., 2019; Atav Ates et al., 2019; Khandelwal et al., 2022; Khatri et al., 2022) assessed post-operative pain levels at the 24-hour interval were consistent with those at 6 and 12 h. Resin-based sealers consistently showed slightly higher pain scores than bioceramic sealers. Importantly, the analysis found no statistically significant difference in pain scores between the two groups (resin-based and bioceramic root canal sealers) as indicated by the obtained *p*-value (*p* > 0.05).

Patients who have undergone root canal therapy could experience pain at 48 h of post operation, which could indicate delated pain or complications. The postoperative pain after 48 h of operation was assessed in four studies (Graunaite et al., 2018; Fonseca et al., 2019; Khandelwal et al., 2022; Khatri et al., 2022). The bioceramic sealers were consistently reported resulting slightly higher pain scores than resin-based sealers. However, based on the statistical analysis with a *p*-value greater than 0.05, it was determined that there was no statistically significant distinction between the resin-based and bioceramic root canal sealers.

The results showed that the analgesics were used in conjunction with root canal sealers to manage post-operative pain. A meta-analysis conducted examined the use of analgesics at 24 h after root canal therapy involving resin-based and bioceramic-based sealers. Data from four studies (Graunaite et al., 2018; Fonseca et al., 2019; Atav Ates et al., 2019; Tan et al., 2021) analyzed revealed that patients receiving resin-based sealers were 1.89 times more likely to require analgesics compared to those with bioceramic-based sealers. However, there was notable variability in the need of analgesics among the studies.

Two of the nine selected studies concluded that pain after the use of bioceramic sealers was lower than that after the use of resin-based sealers (Atav Ates et al., 2019; Khandelwal et al., 2022). The other seven studies (Graunaite et al., 2018; Fonseca et al., 2019; Ferreira de et al., 2020b; Sharma et al., 2020; Tan et al., 2021; Shim, Jang & Kim, 2021; Khatri et al., 2022) concluded that there was no difference between postoperative pain after the use of either bioceramic or resin-based sealers. The results align with the latter group of studies.

The different approaches and methods used by the included studies present one possible research constraint. This might add heterogeneity into the analysis and compromise the validity and generalizability of the findings. Furthermore, the potential for publication bias and the dependence on published research may have an effect on the overall results and distort the conclusions. The generalizability of the results may be further limited by variations in participant demographics and cultural characteristics, as well as in the definition and measurement of postoperative pain.

These limitations might be addressed by subgroup analyses to account for methodological variations. Sensitivity analysis and meta-regression are two further tools for managing heterogeneity. A thorough search approach that includes unpublished studies and grey literature, in addition to statistical measures like Egger’s test and funnel plots. It is essential to take cultural norms and healthcare systems into account when adapting conclusions to various populations and circumstances.

## Conclusions

The pain levels for bioceramic sealers were less than resin-based sealers till 24 h post-operatively. However, after 48 h, the pain levels in the bioceramic group were greater than the resin-based sealers. The findings of this systematic review and meta-analysis suggest that postoperative pain levels with bioceramic sealers had no significant difference from those reported after the use of resin-based sealers.

##  Supplemental Information

10.7717/peerj.18198/supp-1 Supplemental Information 1 PRISMA checklist

10.7717/peerj.18198/supp-2 Supplemental Information 2 Rationale

## Additional Information and Declarations

### Data Availability

The following information was supplied regarding data availability: This is a systematic review/meta-analysis.
