# Supplementary material for: Effect of bioceramic-based and resin-based sealers on postoperative discomfort following root canal therapy: a systematic review and meta-analysis
Source: PeerJ. 2024 Oct 31;12:e18198. doi: 10.7717/peerj.18198 (PMC11531739; doi:10.7717/peerj.18198)
Supplement: Supplemental Information 2 [file peerj-12-18198-s002.docx]

**Rationale**

The ever-present need to enhance endodontic procedures—specifically, root canal therapy—to maximize patient comfort and treatment efficacy is the driving force behind this systematic review and meta-analysis. The foundation of treating root canal infections is root canal therapy, and the choice of obturation materials can have a significant effect on the course of treatment. After endodontic procedures, postoperative pain is a common problem whose management is essential to patient contentment and overall health.

Postoperative pain can be caused by a variety of causes, such as bacterial extrusion, inflammation, and physical damage sustained during therapy. Furthermore, the patient's comfort may be impacted by the root canal sealer selection. Resin-based sealers have strong sealing qualities, but because of things like cytotoxicity and residual monomer release, they might cause more pain after surgery. Conversely, bioceramic root canal sealers have significant sealing capacity, biocompatibility, and antimicrobial characteristics, which may result in decreased levels of discomfort following surgery.

Owing to the importance of this subject, earlier research on the subject has produced inconsistent findings about the connection between postoperative discomfort and root canal sealers. While some studies show no discernible difference between resin-based and bioceramic sealers, others raise the possibility that resin-based sealers might increase postoperative pain. In order to shed light on this issue, a thorough synthesis of the available data is therefore required.

By assessing and contrasting the effects of bioceramic-based and resin-based sealers on postoperative pain following root canal therapy, this systematic review and meta-analysis seek to close this knowledge gap. This study aims to offer evidence-based suggestions for the choice of root canal sealers in actual endodontic treatment by combining data from pertinent clinical research.

The present work makes a significant addition to the field by conducting an extensive review of the literature, which includes a broad spectrum of studies released between January 2000 and August 2022. Through the use of a strict methodology and analysis, this study provides information on the relative effectiveness of resin-based and bioceramic sealers in treating postoperative pain. This information can help dentists make well-informed judgments when selecting obturation materials.

This study contributes to the body of knowledge by offering current and thorough evidence on the subject, taking into account previously published relevant findings, such as other meta-analyses and systematic reviews. This analysis improves patient care and treatment results in endodontics by consolidating and evaluating data from several research to better understand how various root canal sealers affect postoperative pain.
